# Hypertensive Disorders of Pregnancy in Saudi Arabia: Evaluating Maternal and Neonatal Risks, Outcomes, and Aspirin Prophylaxis: A Review Article

**DOI:** 10.7759/cureus.68737

**Published:** 2024-09-05

**Authors:** Tameem M Jayousi, Magdeldin G Taha, Sara M Jaan, Afrah F Aljabri, Samaher I Banaji, Raha Z Ishqi

**Affiliations:** 1 Obstetrics and Gynecology, Taibah University Faculty of Medicine, Madinah, SAU; 2 Internal Medicine, Taibah University Faculty of Medicine, Madinah, SAU

**Keywords:** aspirin prophylaxis, eclampsia, high risk pregnancy, hypertensive disorders during pregnancy, pre-eclampsia prophylaxis, saudi arabia

## Abstract

Hypertensive disorders during pregnancy, including pre-eclampsia and eclampsia, pose significant risks to both maternal and neonatal health. This review article evaluates the prevalence, maternal and neonatal outcomes, and the efficacy of aspirin prophylaxis in managing these conditions in Saudi Arabia. Utilizing data from multiple retrospective studies and recent guidelines, we highlight the regional variations in the outcomes of hypertensive disorders of pregnancy. Severe complications such as Hemolysis, Elevated Liver enzymes and Low Platelets (HELLP) syndrome occurred in 6.6% of cases, while eclampsia was reported in 6.7% of cases. Cesarean sections were notably high, with rates reaching up to 79% among affected pregnancies. Maternal risk factors identified include chronic hypertension (prevalence 17%), diabetes (ranging from 10.4% to 26.3%), and advanced maternal age. Neonatal complications often involve preterm birth, reported in 26.5% to 26.7% of cases, intrauterine growth restriction (ranging from 15.7% to 25%), and increased NICU admissions, reported in 2.4% of cases. No data were found in the included studies to evaluate the prophylactic use of low-dose aspirin in reducing the incidence of pre-eclampsia or improving fetomaternal outcomes. Despite the effectiveness of aspirin, awareness and implementation of prophylaxis guidelines remain suboptimal among healthcare providers in Saudi Arabia. A national survey revealed that only a fraction of obstetrical care providers were fully knowledgeable about aspirin prophylaxis guidelines. This review underscores the necessity for enhanced educational programs and standardized guidelines to improve maternal and neonatal outcomes in hypertensive pregnancies within the region.

## Introduction and background

Pre-eclampsia is a hypertensive disease related to pregnancy, defined as a systolic blood pressure of 140 mm Hg or more, or diastolic blood pressure of 90 mm Hg or more on at least two occasions four hours apart after 20 weeks of gestation in a woman with previously normal blood pressure. Systolic blood pressure of 160 mm Hg or more, or diastolic blood pressure of 110 mm Hg or more, indicates severe hypertension. Proteinuria criteria include: proteinuria of 300 mg or more per 24-hour urine collection (or this amount extrapolated from a timed collection). A dipstick reading of 2+ is used only if other quantitative methods are not available [1].

Many factors contribute to the severity of pre-eclampsia, including blood pressure level, the presence of proteinuria, and other clinical manifestations of end organ damage. Pre-eclampsia affects maternal and fetal health, leading to high maternal morbidity and mortality, fetal growth restriction (FGR), preterm birth, stillbirth, and long-term complications including cardiovascular diseases in both mother and infant, such as neurodevelopmental disorders and bronchopulmonary dysplasia [2]. Globally, the percentage of pre-eclampsia among pregnancies is 3-5% [3]. In Saudi Arabia, recent studies estimate the incidence of hypertensive disorders of pregnancy to affect 2.4% of pregnant women, of whom 55% are diagnosed with pre-eclampsia. These findings underscore the importance of early interventions and aspirin prophylaxis in reducing the risks associated with pre-eclampsia [2].

According to systematic reviews of randomized studies, pre-eclampsia risk can be considerably decreased by using low-dose aspirin during pregnancy [4]. In 2013, American College of Obstetricians and Gynecologists (ACOG) issued the Task Force Report "Hypertension in Pregnancy," which recommended low-dose aspirin in the late first trimester for pregnant women who have a history of early-onset pre-eclampsia and preterm delivery at less than 34 weeks of gestation, or for a woman who developed pre-eclampsia more than once in her previous pregnancies [5]. In July 2018, the ACOG Committee on Obstetric Practice Society for Maternal-Fetal Medicine recommended low-dose aspirin (81 mg/day) prophylaxis in women at high risk of pre-eclampsia, started between 12 weeks and 28 weeks of gestation (optimally before 16 weeks) and continued daily until delivery. Additionally, women with more than one of the several moderate risk factors for pre-eclampsia should take aspirin as prophylaxis. In the absence of risk factors for pre-eclampsia, aspirin prophylaxis is not recommended for women with a prior unexplained stillbirth, FGR, spontaneous preterm birth, or early pregnancy loss [6]. In September 2021, the U.S. Preventive Services Task Force (USPSTF) released revised guidelines and recommendations regarding moderate-risk factors, more specifically, if a person has more than one moderate risk factor, the USPSTF advises to start low-dose aspirin. The USPSTF also included "in vitro conception" as a new moderate-risk factor [7].

In 2021, the WHO guidelines strongly recommended low-dose acetylsalicylic acid (75 mg aspirin per day) to prevent pre-eclampsia in women who are moderately or highly predisposed to the condition. Women are considered to be at moderate risk of developing pre-eclampsia if they have any two of the following risk factors: primiparity, family history of pre-eclampsia, age greater than 40 years, or multiple pregnancies; and at high risk of developing pre-eclampsia if they have one or more of the following risk factors: diabetes, chronic or gestational hypertension, renal disease, autoimmune disease, positive uterine artery Doppler, previous history of pre-eclampsia, or previous fetal or neonatal death associated with pre-eclampsia [8]. The National Institute of Health and Care Excellence (NICE) also recommends that pregnant women at increased risk of pre-eclampsia at the booking appointment are offered a prescription of 75 mg to 150 mg of aspirin to take daily from 12 weeks until birth [9].

Using low-dose aspirin as prophylaxis for pre-eclampsia does not increase bleeding complications during pregnancy and delivery, and the same findings were applied to the risk of fetal congenital anomalies [10-12]. A recent randomized controlled trial (RCT) in 2016 studied aspirin-related symptoms in treated women and found 74% compared to 73% in the placebo arm (p = 0.65), and at least one emergency care visit in 17% versus 16% in placebo (P = 0.76). Vaginal bleeding was reported more in the low-dose aspirin arm at 22% compared with 17%, P = 0.02. Stillbirths, neonatal deaths, and neonatal birth defects were similar between treatment arms [13]. Other reviews found no increase in perinatal deaths due to persistent pulmonary hypertension in the neonates of women treated with aspirin [10,12,14].

The Cochrane meta-analysis in 2019 did not find an increased risk of neonatal intracranial hemorrhage or other neonatal hemorrhagic complications associated with the maternal ingestion of low-dose aspirin during the third trimester. Preterm delivery (16 fewer per 1000 treated), small-for-gestational-age babies (7 fewer per 1000 treated), pre-eclampsia (16 fewer per 1000 treated women), and fetal or neonatal death (5 fewer per 1000 treated), and the result was a 20 percent reduction in births with major unfavorable outcomes [10].

The systematic review on aspirin for the prevention of preeclampsia shows that aspirin significantly reduces the risk of perinatal death, preeclampsia, and spontaneous preterm birth before 34 weeks. Aspirin also increases mean birth weight by 130 grams without increasing the risk of placental abruption [14].

When aspirin was started at ≤16 weeks gestation, there was a significant reduction and a dose-response effect for the prevention of preeclampsia, severe preeclampsia, and fetal growth restriction, with higher dosages of aspirin being associated with greater reductions in these outcomes [15].

In Saudi Arabia, hypertensive disorders have been a significant concern for pregnancy outcomes. Various studies have been conducted to evaluate certain aspects of hypertensive disorders of pregnancy. Hussein MM et al. [16] conducted a retrospective analysis of 135 cases of hypertension in pregnancy, focusing on the presentation, management strategies, and outcomes associated with these conditions. Al-Ghamdi SM et al. [17] provided insights into their medical classification and associated risks to both mothers and babies. Subki AH et al. [18] investigated the prevalence, risk factors, and fetal and maternal outcomes of hypertensive disorders in pregnant women in Western Saudi Arabia. Sobande AA et al. [19] focused on severe pre-eclampsia and eclampsia in the southwest region of Saudi Arabia, providing information on the incidence, management, and outcomes of these severe forms of pregnancy-related hypertension. Al Arfaj AS et al. [20] investigated pregnancy outcomes in 396 pregnancies in patients with systemic lupus erythematosus (SLE) in Saudi Arabia, highlighting the association between autoimmune disorders and pregnancy-related hypertensive disorders. Tameem J et al. [21] conducted a cross-sectional study in Madinah, Saudi Arabia, focusing on the effectiveness of low-dose aspirin in preventing pre-eclampsia and its complications. The study found that low awareness and knowledge of pre-eclampsia among Saudi women were evident, emphasizing the need for increased education on this condition. Alghamdi A et al. [2] presented key findings and conclusions on the prevention of pre-eclampsia in Saudi Arabia, emphasizing the importance of education, standardized guidelines, and awareness in improving pre-eclampsia prevention strategies to reduce associated maternal and fetal morbidity and mortality.

These studies provide a comprehensive picture of the epidemiology, risk factors, management strategies, and maternal-fetal outcomes associated with hypertensive disorders during pregnancy in various regions of Saudi Arabia. They highlight the significant regional variations in the presentation and outcomes of these conditions.

## Review

Literature search, screening, and selection

This study was conducted in accordance with the Preferred Reporting Items for Systematic Reviews and Meta-Analyses (PRISMA) guidelines, ensuring a consistent and reproducible methodology in our systematic review and meta-analysis [22]. A comprehensive search was conducted across multiple databases, including PubMed, Scopus, and Cochrane Library, up to 2024 to capture a broad spectrum of evidence within the Saudi Arabian context. Keywords and MeSH terms such as 'hypertensive disorders during pregnancy,' 'high-risk pregnancy,' 'aspirin prophylaxis,' 'pre-eclampsia prophylaxis,' 'eclampsia,' and 'Saudi Arabia' were combined using Boolean operators to refine the search. The search was restricted to studies published in English to ensure relevance and applicability to the Saudi Arabian healthcare setting.

The inclusion criteria were defined to select studies that specifically addressed hypertensive disorders of pregnancy (HDP) and/or aspirin use in the prevention of pre-eclampsia, focusing on populations in Saudi Arabia. The exclusion criteria included studies not related to pre-eclampsia, eclampsia, hypertension in pregnancy, or risks for pre-eclampsia; those conducted outside the Kingdom of Saudi Arabia (KSA); questionnaire-based studies; studies not published in English; case reports; and reviews.

**Figure 1:**
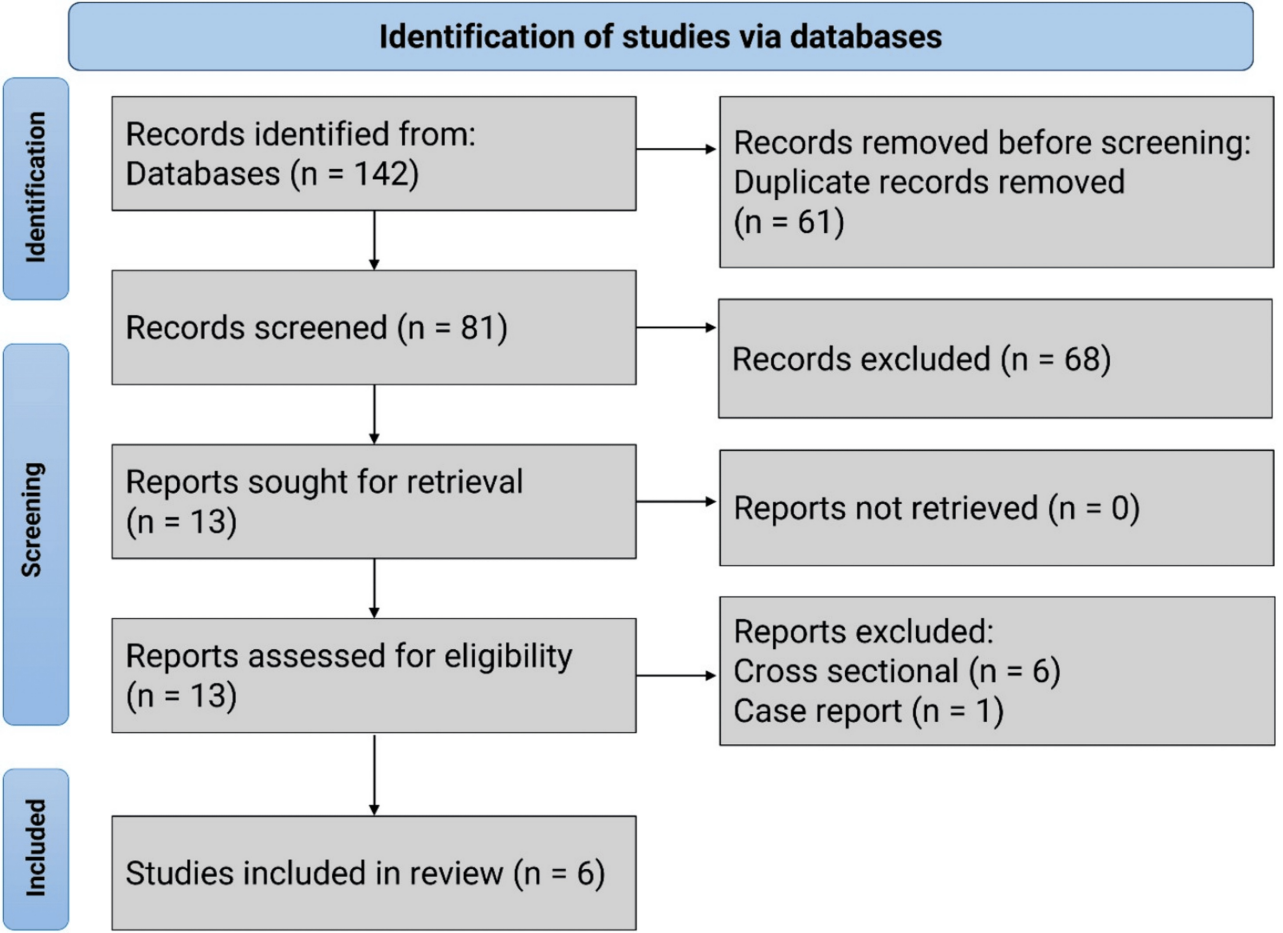
Flowchart of the review on hypertensive disorders of pregnancy in Saudi Arabia.

The preliminary search identified 142 studies. After removing 61 duplicates, 81 studies remained. An additional 68 records were excluded for containing irrelevant data or outcomes, leaving 13 records. Six studies were excluded because they were cross-sectional questionnaire-based studies, and one was excluded as it was a case report. Ultimately, six studies fulfilled the inclusion criteria and were included in the review. No RCTs were identified during the search process (Figure 1).

The included studies

**Figure 2:**
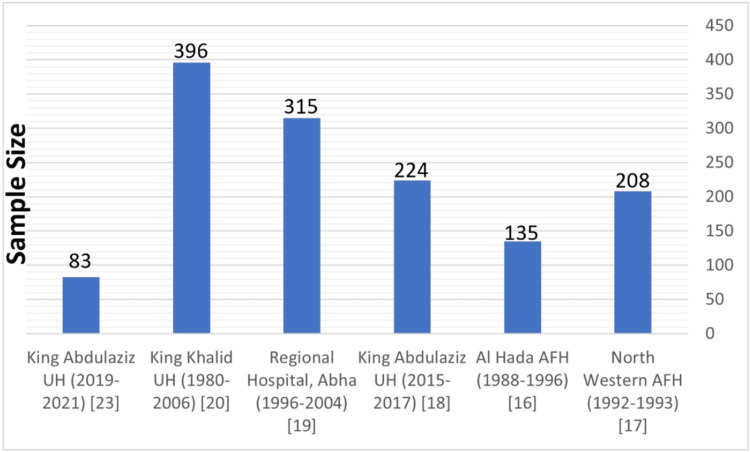
Sample size for the included studies.

The study by Mousa A et al. [23] was carried out at King Abdulaziz University Hospital (KAUH) in Jeddah, Saudi Arabia, and involved 83 patients (with an average age of 32 years). The study examined the feto-maternal-related outcomes of pre-eclampsia patients admitted to the hospital between 2019 and 2021 using descriptive statistical methods. Sobande AA et al. [19] examined 32,000 maternities at Abha General Hospital over 10 years, including 18 cases of eclampsia and 297 cases of severe pre-eclampsia, using multiple regression analysis to identify risk factors. The purpose of the Hussein MM et al. [16] study was to analyze the presentation, management, and outcomes of 135 cases observed at Al Hada Armed Forces Hospital in Taif, Saudi Arabia, over nine years. The study by Al-Ghamdi SM et al. [17] at the North Western Armed Forces Hospital in Tabuk reviewed 208 patient charts, focusing on the prevalence, perinatal mortality, and classification from May 1992 to December 1993. Subki AH et al. [18] at King Abdulaziz University Hospital included 9493 women who delivered between January 2015 and June 2017, focusing on 224 women with HDP. Lastly, Al Arfaj AS and Khalil N [20] at King Khalid University Hospital, Riyadh, analyzed 396 pregnancies in 176 women with SLE from 1980 to 2006, examining the impact of SLE flare, treatment, and other factors on pregnancy outcomes (Table 1, Figure 2).

**Table 1:** Demographic distribution of the included studies. SLE: Systemic lupus erythematosus.

Study Title	Design	Year of the Study	Study Setting	Sample Size	Main outcomes Studied
Hypertensive Disorders of Pregnancy: Prevalence, Classification and Adverse Outcomes in Northwestern Saudi Arabia [17].	Retrospective chart review	1992-1993	Northwestern Armed Forces Hospital, Tabuk, Saudi Arabia	208	Prevalence of hypertensive disorders, maternal and perinatal mortality, classification of hypertension types
Hypertensionin pregnancy: presentation, management and outcome - a retrospective analysis of 135 cases [16].	Retrospective analysis	1988-1996	Al Hada Armed Forces Hospital, Taif, Saudi Arabia	135	Types of hypertensions, maternal and fetal outcomes, treatment efficacy
Prevalence, Risk Factors, and Fetal and Maternal Outcomes of Hypertensive Disorders of Pregnancy: A Retrospective Study in Western Saudi Arabia [18].	Retrospective study	2015-2017	King Abdulaziz University Hospital, Jeddah, Saudi Arabia	224	Prevalence and risk factors of hypertensive disorders, and maternal and fetal outcomes
Severe pre-eclampsia and eclampsia in Abha, the southwest region of Saudi Arabia [19].	Retrospective study	1996-2004	Regional hospital, Abha, Saudi Arabia	315	Prevalence, maternal and neonatal complications from severe pre-eclampsia and eclampsia
Pregnancy outcome in 396 pregnancies in patients with SLE in Saudi Arabia [20].	Retrospective study	1980-2006	King Khalid University Hospital, Riyadh, Saudi Arabia	396	Pregnancy outcomes, impact of hypertension management on fetal and maternal health
Maternal and Fetal Outcomes of Preeclampsia with and Without Severe Features in King Abdulaziz University Hospital, Jeddah, Saudi Arabia: A Retrospective Study [23].	Retrospective study	2019-2021.	King Abdulaziz University Hospital in Jeddah, Saudi Arabia.	83	feto-maternal-related outcomes in terms of complications

The excluded studies

Describing excluded studies enhances the transparency of the review process, demonstrating that a comprehensive search was conducted and providing insight into the selection criteria used. It highlights the methodological rigor of the review by showing that only studies meeting specific criteria were included, ensuring the reliability and validity of the conclusions drawn. Discussing excluded studies also provides educational value to readers and researchers, informing them about common pitfalls and guiding future studies in the field. Additionally, it offers a broader contextual understanding of the research landscape, illustrating the diversity of studies conducted and the reasons for their exclusion. Moreover, the excluded studies delivered good data regarding maternal risk factors for HDP because they were questionnaire-based targeting mothers. This practice strengthens the findings of the included studies by supporting the narrative that they contain the most relevant, high-quality data, thereby reinforcing the review's conclusions.

The study done by Tameem J et al. [21] studied the safety and efficacy of low-dose aspirin in preventing pre-eclampsia and its associated complications in pregnant women in the Al Madinah region. Applying a cross-sectional design, the research involved a sample of 700 participants through an electronic questionnaire disseminated via social media. The findings reveal that low-dose aspirin effectively reduces the severity and complications of pre-eclampsia in 32.1% of the cases, yet it shows no significant effect in 49.3% of the patients. Notably, the study reports no increase in hemorrhagic complications, such as placental abruption, postpartum hemorrhage, or mean blood loss, due to aspirin use during pregnancy. However, the research presents several contradictions, gaps, and inconsistencies that necessitate careful consideration. Firstly, the conclusion that low-dose aspirin may not serve as an effective prophylactic measure for pre-eclampsia in some cases stands in contrast to the WHO's guidelines, which endorse low-dose aspirin for women at high risk of the condition. This discrepancy can result from the study's exclusive emphasis on the Medina context, which might restrict its generalizability to other communities. The study's methodological limitations, including a relatively small sample size and the exclusive collection of data from a single center, raise concerns about the generalizability of its outcomes. The methodology's reliance on social media and electronic questionnaires for data collection might also introduce selection bias, potentially directing the sample toward certain demographics and influencing the reported effectiveness of aspirin prophylaxis. In summary, the study provides insightful information about the possible role of low-dose aspirin in reducing the severity of pre-eclampsia in a particular Saudi Arabian setting, but its inconsistencies with accepted practices highlight the need for additional, more systematic research. The study by Alghamdi A et al. [2] reveals a significant knowledge gap among obstetrical care providers about aspirin prophylaxis, including its dosage, timing, and guidelines. Although maternal fetal medicine specialists and physicians in teaching hospitals showed better knowledge, overall awareness of risk factors and aspirin use was insufficient. The study emphasizes the need for enhanced educational programs and continuous medical training to improve prevention practices. It also highlights the importance of aspirin in managing high-risk pregnancies but notes the study's limited participation rate, potentially affecting the findings' applicability. They recommend initiating awareness programs aimed at both healthcare providers and patients to ensure the early detection of high-risk pregnancies and encourage adherence to aspirin prophylaxis, emphasizing pre-eclampsia as a critical public health problem. Also, the study by Bahkali N [24] was a cross-sectional study conducted from September 2022 to December 2022 in the Western Region of Saudi Arabia. It included 541 women, aged 18-50, not involved in the medical field. The data were collected using a validated and reliable questionnaire distributed via social media, shopping centers, outpatient clinics, and inpatient wards of King Abdulaziz University Hospital. The study conducted by Gari A et al. [25] aimed to assess the awareness and understanding of pre-eclampsia among women in Makkah, Saudi Arabia. Conducted between June and July 2022 with 378 participants, a validated online questionnaire was used to evaluate their knowledge of various aspects of pre-eclampsia. The findings revealed a remarkably low level of awareness, with only 4% of the participants demonstrating a good knowledge of the condition. This underscores the critical need for effective health education programs to improve knowledge and awareness of pre-eclampsia among women in the region. The study by Radwan A et al. [26] conducted from June to August 2023, evaluated the understanding of pre-eclampsia among 1169 Saudi women aged 18-49. This cross-sectional study revealed that a significant portion of the participants had inadequate knowledge about pre-eclampsia, with only 11.3% displaying adequate understanding. The research highlighted the need for targeted educational programs to raise awareness and improve knowledge about pre-eclampsia among Saudi women, emphasizing the importance of early diagnosis and care throughout pregnancy to prevent maternal and fetal complications. The study by Alghanim KK et al. [27] was a case report study describing a 31-year-old woman with a history suggestive of obstetric antiphospholipid syndrome (APS), characterized by recurrent miscarriages and intrauterine fetal death. The study was conducted at the Rheumatology Clinic of King Fahad Military Medical Complex, Dhahran, Saudi Arabia. This study was excluded from the analysis. The last one, Alghadeer S et al. [28] was a cross-sectional study conducted in 2021 over four months, targeting Saudi adults using online questionnaires. This study aimed to evaluate the prevalence of aspirin use and to identify the demographic and clinical characteristics of Saudi users (Table 2).

**Table 2:** Demographic distribution of the excluded studies.

	Study Title	Design	Year of the Study	Study Setting	Sample Size	Main outcomes Studied
1	Aspirin as a Prophylaxis in Pre-Eclampsia and Its Complications in Medina, Saudi Arabia [21].	Cross-sectional survey	2019	Al Madinah region, Saudi Arabia	700	Safety and efficacy of low-dose aspirin in preventing pre-eclampsia and its complications
2	Prevention of Pre-eclampsia in Saudi Arabia Knowledge about Risk Factors and Aspirin Use among Obstetrical Care Physicians - A National Survey [2].	Cross-sectional survey	2024	Saudi Arabia	160	Knowledge gap among obstetrical care providers about aspirin prophylaxis
3	Awareness of Pre-eclampsia and Measurement of Risk Factors in Women from the Western Region of Saudi Arabia: A Cross-sectional Study [24].	Cross-sectional survey	2022	Western Region of Saudi Arabia	541	Awareness and measurement of risk factors of pre-eclampsia
4	Level of knowledge on pre-eclampsia symptoms, complications, and risk factors among women in Saudi Arabia: A cross-sectional study [25].	Cross-sectional survey	2022	Makkah, Saudi Arabia	378	Knowledge of pre-eclampsia symptoms, complications, and risk factors
5	Awareness and Knowledge of Pre-eclampsia Among Saudi Women of Reproductive Age [26].	Cross-sectional survey	2023	Saudi Arabia	1169	Knowledge about pre-eclampsia
6	Seronegative Obstetric Antiphospholipid Syndrome Finally Treated Successfully [27].	Case report	2022	King Fahad Military Medical Complex, Dhahran, Saudi Arabia	1	Case of obstetric antiphospholipid syndrome
7	Aspirin Use among Saudi Adults: The Prevalence and Users' Characteristics [28].	Cross-sectional survey	2021	Saudi Arabia	771	Prevalence of aspirin use and characteristics of users

Quality assessment of included studies

Due to the nature of the studies, which are primarily retrospective analyses and reviews of medical records, common risks of bias include selection bias, where participants aren't randomly chosen, potentially making the results unreliable; measurement bias, where retrospective data collection relies on existing records that might be incomplete or inaccurate, leading to misclassification of exposure or outcomes; and confounding variables, where other factors not considered in the study might influence both the exposure and the outcome, making it appear as though there is a direct connection when there isn't. It is good practice to be aware of these limitations and look for studies that acknowledge and attempt to mitigate these biases.

The retrospective analysis by Hussein MM et al. [16] over a nine-year period at a Saudi Arabian hospital captures the complexity of hypertension in pregnancy through a detailed review of 135 cases. While comprehensive in its approach to identifying hypertension using medical records and evaluating maternal and fetal outcomes, the study's comparability is somewhat limited due to the lack of specific adjustments for potential confounders. Additionally, variable follow-up durations postpartum introduce challenges in consistently assessing long-term hypertension outcomes.

Al-Ghamdi SM et al. [17] conducted a retrospective cohort study that provides a detailed analysis of hypertensive disorders in pregnancy, applied reliable classification systems for hypertension, and utilized hospital records for outcome assessment. It accurately identifies the prevalence and types of hypertensive disorders in 208 patients from 6710 deliveries over a 20-month period, ensuring that the hypertensive outcomes of interest are observed within an appropriate timeframe. However, the study's quality is slightly limited by its focus on a military population and the lack of extensive comparability analysis, both of which are critical for improving the generalizability and depth of the findings.

The study by Subki AH et al. [18], conducted at King Abdulaziz University Hospital, offers a moderate-to-high-quality investigation into hypertensive disorders during pregnancy. Analyzing data from 224 women out of 9,493 deliveries over a 2.5-year period, this research not only identifies the prevalence of these disorders but also explores associated risk factors and maternal and fetal outcomes. The use of established criteria for diagnosing hypertensive disorders ensures accurate identification and reliable analysis of outcomes, including maternal complications, delivery modes, and neonatal Apgar scores. The comprehensive follow-up through medical records up until delivery achieves a complete outcome assessment.

The retrospective study by Sobande AA et al. [19], focusing on severe pre-eclampsia and eclampsia in Abha, Saudi Arabia, demonstrates strict methodology for detecting severe conditions using well-defined clinical criteria, as well as a full capture of maternal and neonatal outcomes over a ten-year period. While the study provides valuable insights into the prevalence and impact of these conditions in a specific geographic area, its quality is somewhat limited by the absence of explicit comparability controls.

The study by Al Arfaj AS and Khalil N [20] published in 2010, is notable for its comprehensive examination of pregnancy outcomes among SLE patients over a significant period of 27 years. The statistical analysis applied, including logistic regression, allowed for an in-depth understanding of the influence of various SLE characteristics and treatment modalities on pregnancy outcomes. The extensive sample size and longitudinal design emphasize the study's high quality and contribute to the reliability of its findings. However, the retrospective nature of the study introduces inherent biases concerning data collection accuracy and selection bias, and the fact that the patient population was from a single hospital in Saudi Arabia may limit the generalizability of its findings.

The study by Mousa A et al. [23], utilizing a retrospective design effectively, clearly states its objectives, uses appropriate statistical analyses, and reports its findings comprehensively. However, the lack of sensitivity analysis limits the interpretation of how reliable the findings are under different assumptions or potential biases. Overall, the study contributes valuable knowledge to the field but highlights common challenges in retrospective research that future studies could address more thoroughly.

These evaluations collectively highlight the critical balance between the strengths of the studies, such as extensive sample sizes and long durations, and the inherent biases introduced by their retrospective designs. Such biases include potential issues with data accuracy, selection bias, and limited generalizability, making it difficult to accurately assess pregnancy outcomes and complications in patients with hypertensive disorders.

Discussion

Maternal Risk Factors for HDP

**Figure 3:**
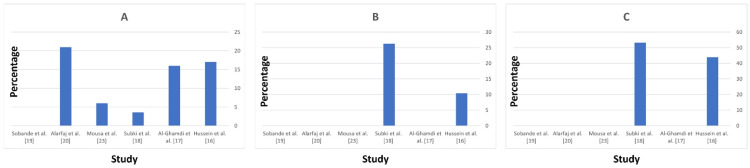
Maternal risk factors for hypertensive disorders of pregnancy by study. (A) History of chronic hypertension; (B) Diabetes mellitus; (C) History of preeclampsia.

The data from the studies highlight several key maternal risk factors for HDP. Chronic hypertension is a significant risk factor with a prevalence ranging from 3.6% (Subki AH et al. [18]) to 21% (Al Arfaj AS and Khalil N [20]), and it notably increases the likelihood of developing superimposed preeclampsia, observed in 47% of cases with pre-existing hypertension (Al-Ghamdi SM et al. [17], Figure 3A). Diabetes mellitus is another critical risk factor, strongly associated with hypertensive disorders, particularly preeclampsia and gestational hypertension, with the highest prevalence reported at 26.3% (Subki AH et al. [18], Figure 3B). A history of preeclampsia is also a significant predictor of recurrence and complications, with personal history reported in 44% (Hussein MM et al. [16]) to 53.3% (Subki AH et al. [18]) of cases, and family history indicating a genetic predisposition in 47.8% (Subki AH et al. [18], Figure 3C). These findings underscore the importance of early screening, monitoring, and management of these risk factors to mitigate adverse maternal and fetal outcomes.

Maternal Complications in Mothers With HDP

**Figure 4:**
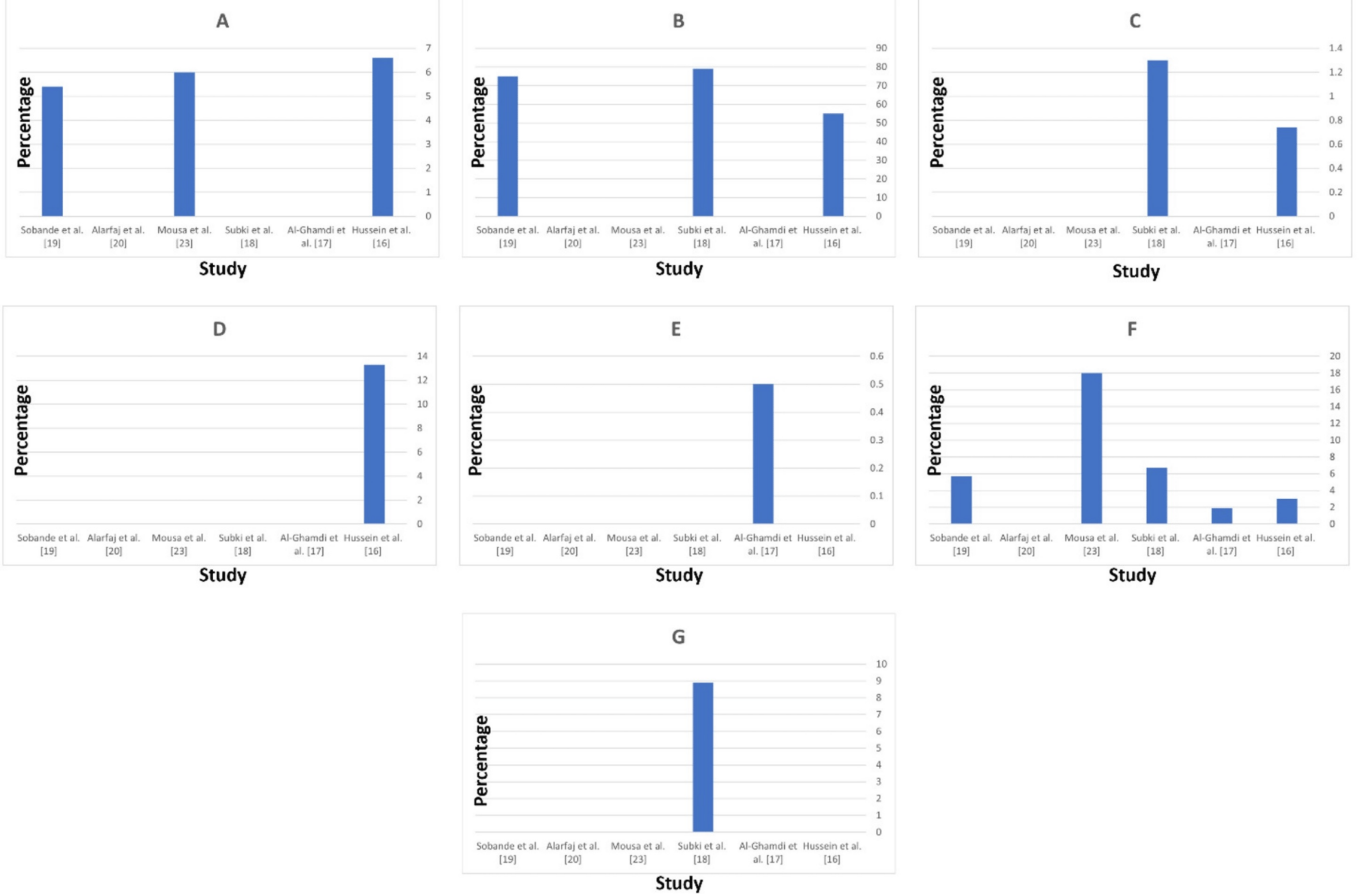
Maternal complications in mothers with hypertensive disorders of pregnancy. (A) HELLP syndrome; (B) Cesarean section; (C) Maternal mortality; (D) Persistent hypertension; (E) Pulmonary edema; (F) Eclampsia; (G) ICU admission.

HELLP syndrome, a severe form of preeclampsia involving hemolysis, elevated liver enzymes, and low platelet count, was reported with varying frequencies. Hussein MM et al. [16] reported a 6.6% prevalence of HELLP syndrome among their study population. Mousa A et al. [23] noted an incidence of 6%, while Sobande AA et al. [19] reported a 5.4% prevalence. Subki AH et al. [18] and Al-Ghamdi SM et al. [17] did not report any cases of HELLP syndrome. These minimal differences highlight the variability in the occurrence of HELLP syndrome across different regions and study populations in Saudi Arabia (Figure 4A).

Cesarean section rates were notably high among women with hypertensive disorders. Subki AH et al. [18] reported a very high rate of 79% for cesarean deliveries, indicating a common intervention in managing complicated pregnancies. Hussein MM et al. [16] also reported a high rate of 55%, while Sobande AA et al. [19] reported 75%. Mousa A et al. [23] did not provide specific data on cesarean section rates. The high rates of cesarean sections reflect the need for immediate delivery to manage severe hypertensive complications (Figure 4B).

Maternal mortality is a critical outcome reflecting the severity of complications. Subki AH et al. [18] reported a maternal mortality rate of 1.3% among women with HDP. Hussein's study [16] reported one case of maternal death, corresponding to a rate of approximately 0.74%. Sobande AA et al. [19] did not report any cases of maternal mortality. Mousa A et al. [23] reported a maternal mortality rate of 0%, indicating effective management of HDP in their study population. This low maternal mortality rate underscores the importance of timely and effective management of HDP (Figure 4C).

Persistent hypertension was specifically reported by Hussein MM et al. [16], with a prevalence of 13.3% among women with hypertensive disorders. This condition requires careful monitoring and management. Hussein's study defined persistent hypertension as hypertension continuing for more than two months after delivery in patients not previously known to have chronic hypertension. Subki AH et al. [18] and other studies did not report specific data on persistent hypertension after delivery (Figure 4D).

Pulmonary edema, a life-threatening condition characterized by fluid accumulation in the lungs, was reported at a very low prevalence. Al-Ghamdi SM et al. [17] reported a prevalence of 0.5%. These low frequencies indicate that while pulmonary edema is a severe complication, it occurs infrequently among women with hypertensive disorders. Hussein MM et al., Subki AH et al. [18], and Mousa A et al. [23] did not report cases of pulmonary edema, suggesting its rarity (Figure 4E).

Eclampsia, a severe complication of preeclampsia characterized by seizures, according to Al-Ghamdi SM et al. [17], occurred in 1.9% of HDP cases in Northwestern Saudi Arabia, with an overall prevalence of 0.06% of all deliveries. Magdi M. Hussein's [16] study reported eclampsia in 4 pregnancies (3% of the total study population). Subki AH et al. [18] reported a 6.7% prevalence of eclampsia among women with HDP. Mousa A et al. [23] reported a prevalence of 18%, but Sobande AA et al. [19] reported a prevalence of 5.7%. These data indicate that eclampsia, although less frequent than preeclampsia, poses significant risks to maternal and fetal health (Figure 4F).

ICU admissions reflect the severity of maternal complications requiring intensive care. Subki AH et al. [18] reported that 8.9% of women with eclampsia were admitted to the ICU. This high rate underscores the severe nature of eclampsia and the need for advanced medical care to manage such cases effectively. Hussein MM et al. [16], Al-Ghamdi SM et al. [17], Sobande AA et al. [19], and Mousa A et al. [23] did not report specific percentages but indicated that severe cases often required ICU care (Figure 4G).

Cerebral hemorrhage is a rare but severe complication. Sobande AA et al. [19] reported one case, which represents 0.3% of the studied population. Other studies reviewed did not report any prevalence rates for cerebral hemorrhage, suggesting its rarity in the studied populations.

Renal failure, another severe complication, was also infrequently reported. Al-Ghamdi SM et al. [17], noted no cases of renal failure among their study participants. The absence of reported cases in other studies, including Hussein MM et al. [16], Sobande AA et al. [19], Subki AH et al. [18], and Mousa A et al. [23], suggests that renal failure is a rare outcome in hypertensive pregnancies.

Heart failure was similarly rare, with Al-Ghamdi SM et al. [17] reported one case during their study period. Hussein MM et al. [16], Sobande AA et al. [19], Subki AH et al. [18], and Mousa A et al. [23] did not report any cases of heart failure. This indicates that, although heart failure is a serious potential complication, it is not commonly observed in pregnancies complicated by hypertensive disorders.

Perinatal and Neonatal Outcomes and Complications

**Figure 5:**
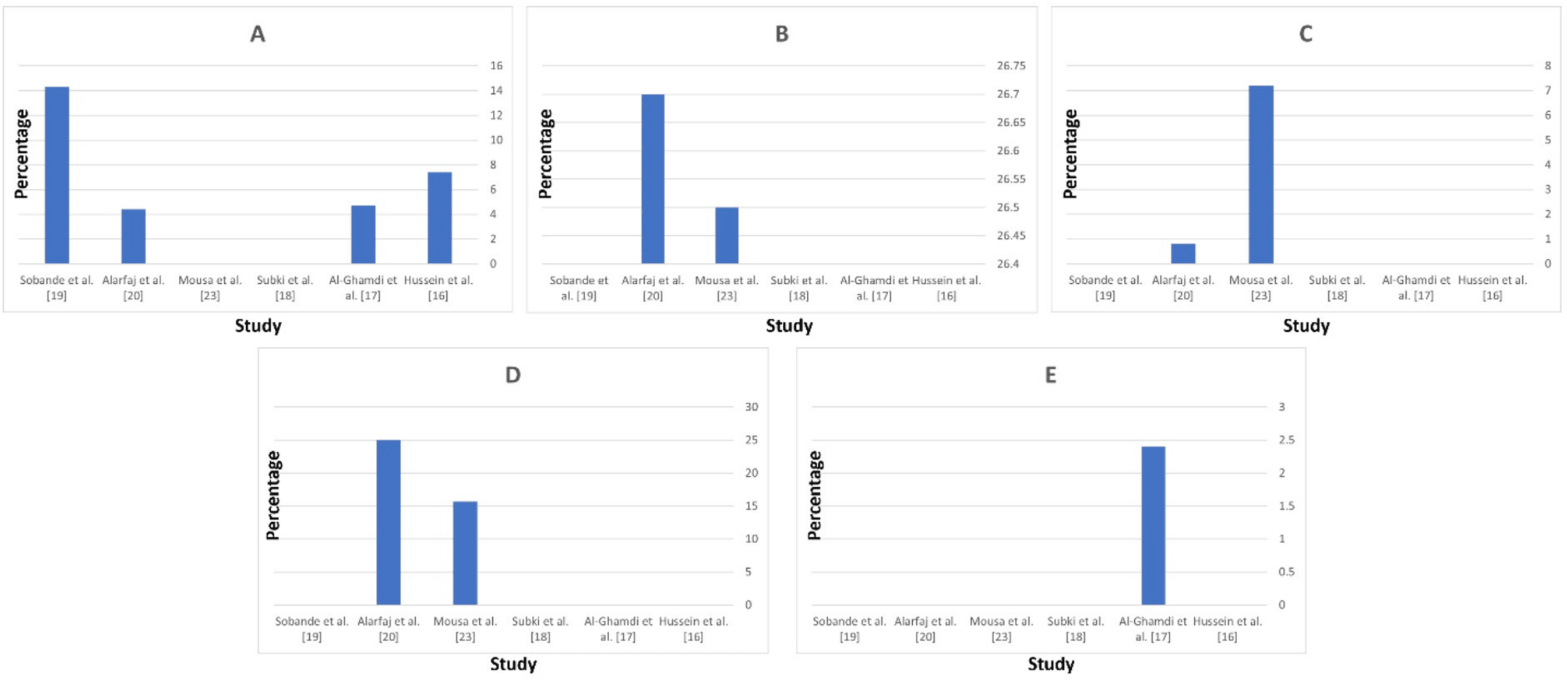
Perinatal and neonatal outcomes and complications. (A) Perinatal mortality; (B) Preterm birth; (C) Neonatal death; (D) Intrauterine growth restriction; (E) Neonatal ICU admission.

The data presents an overview of the perinatal and neonatal outcomes and complications associated with HDP. Perinatal mortality, which includes the death of a fetus or neonate within the first 7 days of life, shows significant variability among studies: Hussein MM et al. [16] reported a rate of 7.4%, Al-Ghamdi SM et al. [17] observed 4.7%, Al Arfaj AS and Khalil N [20] reported 4.4%, and Sobande AA et al. [19] reported the highest rate at 14.3%. These figures highlight the substantial impact of hypertensive disorders on perinatal outcomes, with rates significantly higher than those in the general population in some regions (Figure 5A).

Preterm birth, defined as delivery before 37 weeks of gestation, is another common complication. Subki AH et al. [18] reported a gestational age range of 31.8 ± 3.5 to 34.5 ± 4.1 for HDP but did not report the rate of preterm births due to hypertensive complications. Mousa A et al. [23] observed a slightly lower rate of 26.5%, and Al Arfaj AS and Khalil N [20] reported the highest at 26.7%. This underscores the critical need for managing hypertension in pregnancy to prevent early deliveries and associated neonatal complications (Figure 5B).

Neonatal death, referring to the death of a live-born baby within the first 28 days, also varies significantly: Mousa A et al. [23] found a much higher rate of 7.2%, while Al Arfaj AS and Khalil N [20] noted a rate of 0.8%. These rates emphasize the severe impact of hypertensive disorders on neonatal survival and the importance of early detection and management to improve outcomes (Figure 5C).

Intrauterine Growth Restriction (IUGR), where a fetus does not grow to its expected size, was reported at 15.7% by Mousa A et al. [23] and 25% by Al Arfaj AS and Khalil N et al. [20]. These data highlight the significant impact of hypertensive disorders on fetal growth, necessitating close monitoring and management to decrease adverse outcomes (Figure 5D).

Finally, the need for NICU admission, indicating severe neonatal complications requiring specialized care, was reported at 2.4% by Al-Ghamdi SM et al. [17]. The need for NICU admission underscores the severity of the impact of hypertensive disorders on neonatal health and the importance of providing specialized care to improve outcomes for these infants (Figure 5E).

## Conclusions

These studies highlight the critical need for early detection, continuous monitoring, and specialized healthcare for pregnancy-related complications. Women with preeclampsia, eclampsia, and hypertension face higher risks of adverse maternal and neonatal outcomes, including increased rates of cesarean sections and preterm deliveries. To improve outcomes, especially for preeclampsia and eclampsia, strategies should focus on qualified professionals trained in emergency obstetric care, facilities equipped for blood pressure monitoring, and timely referrals. Enhancing maternal-fetal medicine, screening programs, specialist care, and research investment is essential to reduce risks and improve outcomes on a larger scale.
